# Time-Dependent Endurance Exercise Improves Metabolic Health Through Circadian Rhythm Regulation in Mice

**DOI:** 10.3390/jfmk11020226

**Published:** 2026-06-01

**Authors:** Yanqing Zhou, Qianyun Cheng, Zuoqing Yan, Chao Lu, Bingxuan Hua

**Affiliations:** 1 Department of Physiology and Pathophysiology, School of Basic Medical Sciences, Fudan University, NO. 130, Dongan Road, Xuhui District, Shanghai 200030, China; 23111010101@m.fudan.edu.cn (Y.Z.); qycheng16@fudan.edu.cn (Q.C.); 2 Institute of Bone and Joint Diseases, Fudan University, Shanghai 200032, China; yan.zuoqin@zs-hospital.sh.cn; 3 Department of Orthopedics, Zhongshan Hospital, Fudan University, Shanghai 200032, China

**Keywords:** circadian rhythms, endurance exercise, metabolic health, time-restricted exercise, *clock*^Δ19^ mice, lipid metabolism, insulin sensitivity

## Abstract

**Objectives**: Circadian rhythms regulate key physiological processes, including metabolism and energy balance. Emerging evidence suggests that the timing of physical activity may influence metabolic outcomes. However, how the timing of endurance exercise impacts long-term metabolic health and the role of the circadian clock in this process remain unclear. This study aimed to investigate whether time-dependent endurance exercise improves metabolic health via circadian rhythm regulation. **Methods**: A 12-week endurance exercise protocol was established using wild-type (WT) and circadian-disrupted *Clock*^Δ19^ mice. Mice were assigned to exercise at Zeitgeber time 0 (ZT0) or Zeitgeber time 0 (ZT12), or to sedentary controls. Assessments included rotarod fatigue test, body weight, epididymal fat ratio, fasting blood glucose, serum triglycerides, high-density lipoprotein cholesterol (HDL-C), low-density lipoprotein cholesterol (LDL-C), non-esterified fatty acids (NEFA), intraperitoneal glucose tolerance test (IPGTT), and insulin tolerance test (ITT). **Results**: *Clock*^Δ19^ mice exhibited circadian phase-dependent fatigue susceptibility on the rotarod, particularly at ZT0. Both exercised *Clock*^Δ19^ groups (ZT0 and ZT12) showed significant weight reduction compared to sedentary controls, indicating that endurance exercise may counteracts circadian disruption-induced weight gain independent of timing. In WT mice, evening exercise (ZT12) led to enhanced lipid regulation and better glucose tolerance. These time-dependent benefits were absent in *Clock*^Δ19^ mutants, demonstrating that the full metabolic advantages of exercise require a functional circadian clock. Notably, endurance training also partially restored serum HDL-C levels in *Clock*^Δ19^ mice, suggesting compensatory metabolic responses. **Conclusions**: Aligning endurance exercise with the body’s internal clock provides greater metabolic benefits than untimed exercise. The circadian clock is essential for time-dependent improvements in glucose and lipid metabolism, although some beneficial effects occur independently of a functional clock.

## 1. Introduction

Circadian rhythms—endogenous ~24-h cycles regulating physiology and behavior—are essential for maintaining metabolic health [1]. These rhythms coordinate processes such as feeding [2], physical activity, and energy use [3], and are governed by a molecular clock involving core genes like *Clock* and *Bmal1* [4]. Specifically, the CLOCK::BMAL1 heterodimer acts as a master transcriptional factor, directly binding to E-box elements to orchestrate the expression of key metabolic enzymes, such as those governing fatty acid oxidation and mitochondrial respiration in peripheral tissues like skeletal muscle and liver [5]. Disruptions to this system, whether through genetic mutations or environmental misalignment (e.g., shift work), are closely linked to metabolic disorders, including obesity, insulin resistance, and dyslipidemia [6,7]. While exercise is a well-established intervention for improving metabolic outcomes, recent evidence suggests that its benefits may depend on the time of day it is performed [8,9,10], due to interactions with circadian pathways. However, most existing literature focuses primarily on the acute metabolic responses to a single bout of timed exercise [11,12]. The mechanisms by which long-term, chronic, time-specific exercise influences cross-tissue metabolic adaptations—and whether these systemic adaptations fundamentally require a functional autonomous circadian clock—remain unclear [13,14]. Furthermore, while various tissue-specific clocks (e.g., skeletal muscle vs. adipose tissue) respond differently to physical stimuli, how time-specific exercise coordinates these tissue-specific pathways over a sustained period is poorly understood.

The interaction between exercise and the circadian system is increasingly recognized as bidirectional. On one hand, exercise can shift peripheral clocks in tissues such as skeletal muscle; on the other, circadian rhythms influence exercise performance and metabolic responses [13,14]. For instance, rodent studies show daily fluctuations in exercise capacity, linked to changes in energy availability and clock gene expression [14]. However, findings across studies remain inconsistent: some suggest that evening exercise offers greater metabolic benefits in humans, while others report enhanced fat oxidation or insulin sensitivity with morning activity [15]. These discrepancies may stem from differences in study design, species-specific activity patterns, or the timing of food intake. Notably, few studies have rigorously evaluated the long-term effects of time-restricted exercise on systemic metabolism, especially in models with disrupted circadian function, leaving key questions unresolved.

To address these gaps, we examined how three months of endurance exercise performed either in the morning (ZT0) or evening (ZT12) affected metabolic health in wild-type (WT) mice and *Clock*^Δ19^ mutants. The *Clock*^Δ19^ mouse model harbors a dominant-negative deletion of exon 19 in the *Clock* gene, which disrupts transcriptional activation without completely abolishing protein expression, perfectly mimicking the chronic, state-dependent circadian desynchrony and subsequent metabolic syndrome observed in shift-working human populations [16]. We hypothesized that: (1) exercise timing would differentially affect glucose tolerance, lipid metabolism, and insulin sensitivity in WT mice, with greater benefits during their active phase (ZT12); (2) these time-dependent effects would be lost in *Clock*^Δ19^ mice, highlighting the importance of a functional circadian clock; and (3) exercise would still confer metabolic improvements in *Clock*^Δ19^ mice, independent of timing, via alternative regulatory mechanism. To test these hypotheses, we subjected both genotypes to a 12-week timed endurance exercise regimen and subsequently performed comprehensive physiological, metabolic, and transcriptomic assessments, including glucose tolerance testing, systemic lipid profiling, and RNA-sequencing of skeletal muscle.

By integrating genetic, physiological, and molecular data, this study provides new insight into how the timing of physical activity interacts with circadian rhythm. These findings have important implications for personalized exercise strategies, particularly in populations with circadian disruption, such as shift workers or older adults.

## 2. Materials and Methods

### 2.1. Animals

C57BL/6 mice were obtained from the Model Animal Research Center of Nanjing University (Nanjing, China). *Clock*^Δ19^ mice were obtained from Jackson Laboratory (Bar Harbor, ME, USA). Animal experiments were approved by the Fudan University Institutional Animal Care and Use Committee (Approval ID: 20210302-065). Mice were housed in a temperature-controlled room (24 ± 1 °C) with a relative humidity of 50–60%. Prior to the long-term exercise intervention, animals were acclimated to a standard 12h light/12h dark cycle (light on at 8:00 am, light off at 8:00 pm) for two weeks. To investigate the endogenous role of the circadian clock without photic interference, mice were maintained in a constant darkness (DD) environment (0 lux) during the 12 weeks of endurance exercise training. Minimal disruptions required for cage maintenance were handled exclusively under dim red light. To prevent the loss of behavioral rhythms in the absence of light-dark cues, a time-restricted feeding (TRF) schedule was established. Food was manually introduced into the cages daily at the onset of the subjective active phase (ZT12) and completely removed, including any residual food debris, at the onset of the subjective rest phase (ZT0). Animals were randomly assigned to sedentary (SE) or exercise (ZT0 or ZT12) groups.

### 2.2. Treadmill Running

Twelve-week-old male wild-type (WT) and *Clock*^Δ19^ mice (*n* = 5/genotype/group) underwent moderate-intensity endurance training for 15 weeks using a motorized treadmill (Columbus Instruments Exer-3/6, Columbus Instruments, Columbus, OH, USA) equipped with an electric stimulation grid (0.3 mA constant current). Following a 2-week adaptive training protocol (Day 1–7: 5 min at 5 m/min, 0° incline; Day 8–14: 10 min at 10 m/min, 0° incline), mice performed daily running sessions at either ZT0 (dawn, rest phase) or ZT12 (dusk, active onset) under dim red light (<1 lux). Exercise intensity was maintained at 65–70% VO_2_max (calibrated via workload-VO_2_ regression in pilot studies), corresponding to 10 m/min 55 min/day. Exhaustion was defined as failure to maintain running position despite gentle prodding (>10 s on grid) or receiving >100 cumulative electrical stimuli. The treadmill was equipped with an automated shock counter (Columbus Instruments, Columbus, OH, USA). A “shock” was defined as a 0.3 mA constant current delivered through the electric grid for 0.5 s. The endpoint of exhaustion was defined as the mouse receiving 100 cumulative shocks without maintaining running speed (i.e., staying on the grid for >10 s consecutively). The counter reset if the mouse resumed running for >5 s without contacting the grid. Sedentary controls were placed on a stationary treadmill for identical durations. Animals requiring >3 electrical stimulations/session during adaptive training were excluded. All procedures complied with IACUC guidelines.

### 2.3. Rod Fatigue Test

Motor coordination and fatigue resistance were assessed in 12-week-old wild-type (WT) and *Clock*^Δ19^ mice (*n* = 5/group) using an accelerating rotarod (Ugo Basile, Gemonio, VA, Italy). Mice underwent three consecutive trials per day for two consecutive days at ZT0 (rest phase) and ZT12 (activity onset) under dim red light (<1 lux), with 30-min inter-trial intervals. Each trial began at 4 rpm with acceleration to 40 rpm over 300 s (linear ramp mode). Fatigue endpoint was defined as the latency to fall (seconds) or voluntary dismount (>2 consecutive rotations without re-mounting). To prevent injury, a soft bedding cushion was placed beneath the rod. Data from Day 2 trials were averaged for analysis to exclude learning effects. Animals showing freezing behavior (>10 s immobility at start) or repeated jumping (>3 jumps/trial) were excluded.

### 2.4. Serum Biochemical Assay

At the end of the 15-week training period, following a 12-h fast (from ZT0 to ZT12), mice were anesthetized with isoflurane (RWD Life Science, Shenzhen, China). Blood samples were collected via cardiac puncture between ZT12 and ZT13, immediately after the fasting period and before the onset of the dark phase feeding. Serum was separated by centrifugation at 3000 rpm for 20 min at 4 °C, and the supernatant was collected for analysis. Concentrations of LDL-C, HDL-C, triglycerides, total cholesterol, glucose, and NEFA were measured according to manufacturer’s instructions (Nanjing Jiancheng Bioengineering Institute, Nanjing, China).

### 2.5. Insulin Sensitivity and Glucose Tolerance Test

To evaluate systemic glucose regulation, insulin sensitivity and tolerance tests were conducted after the 12-week endurance exercise protocol. Following a 12-h fast (from ZT0 to ZT12), mice were subjected to the tests at ZT12 (onset of the active phase). For the insulin sensitivity test (IST), mice received a subcutaneous injection of insulin (0.6 U/kg; insulin from Sigma-Aldrich, St. Louis, MO, USA), with blood glucose levels monitored at 0, 15, 30, 60, 90, and 120 min via tail bleed. Similarly, an intraperitoneal glucose tolerance test (IPGTT) was performed by administering glucose (2 g/kg; D-glucose from Sigma-Aldrich, St. Louis, MO, USA) intraperitoneally, followed by blood glucose measurements at identical time intervals.

### 2.6. Statistical Analysis

Statistical analysis was performed using GraphPad Prism 9.5.0 (GraphPad Software Inc., Boston, MA, USA). Results are expressed as means ± SDs, and statistical significance was calculated using Student’s *t*-test. Alternatively, for comparisons involving more than 2 groups, one-way analysis of variance (ANOVA) with Tukey’s multiple comparisons test was used to assess the differences between the groups. Statistical significance was defined as the conventional *p* value of <0.05.

## 3. Results

### 3.1. Chronograph of 12-Week Endurance Exercise Protocol with ZT0 and ZT12 Training Cohorts

**Figure 1:**
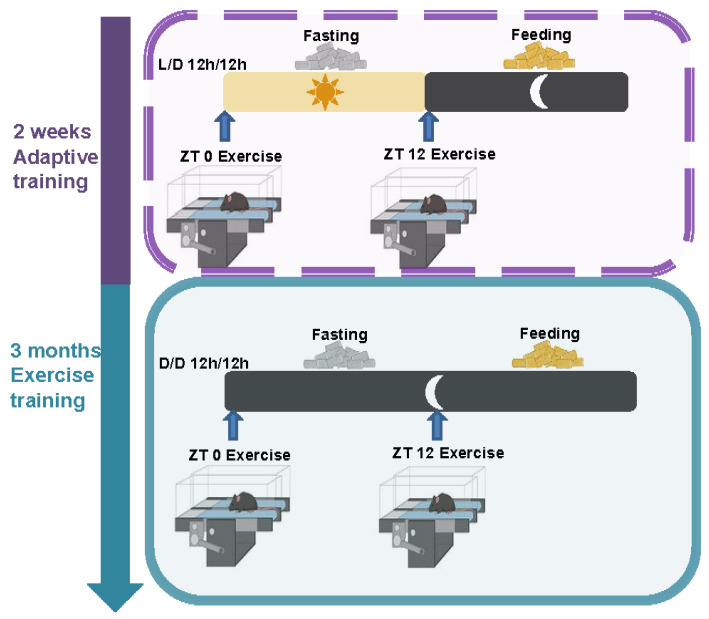
Diagram of the long-term endurance exercise schedules.

As illustrated in Figure 1, 12-week-old *Clock*^Δ19^ and wild-type (WT) mice were randomly assigned to one of three groups: (1) ZT0-EX, initiating exercise at the onset of the light phase (zeitgeber time 0); (2) ZT12-EX, beginning exercise at the onset of the dark phase (zeitgeber time 12); and (3) sedentary controls (SE) subjected to the same handling without exercise. After completing the three-month exercise regimen, mice were humanely euthanized using an isoflurane overdose at time points matched to their respective zeitgeber phases.

### 3.2. Clock^Δ19^ Mice Exhibit Circadian Phase-Dependent Fatigue Susceptibility on Rotarod but Unaltered Treadmill Endurance

**Figure 2:**
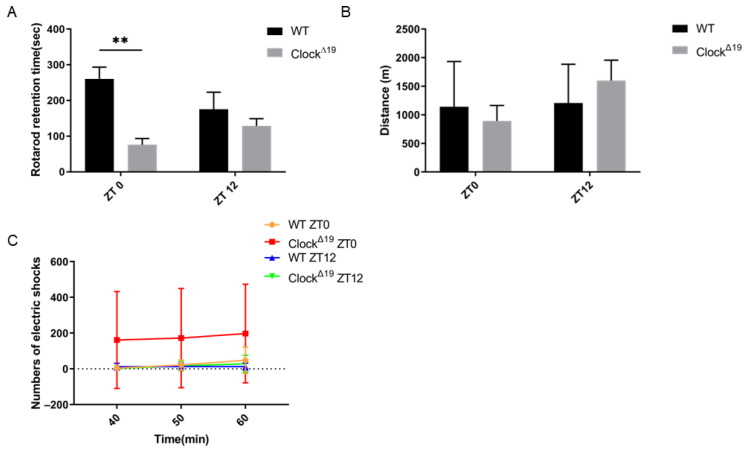
*Clock*^Δ19^ mutation disrupts circadian exercise regulation. (**A**) Rotarod retention time(sec) at 20 rpm fixed speed. (**B**) Total running distance until 100th shock in WT vs *Clock*^Δ19^. (**C**) Number of electric shocks received within specific time intervals. The data are presented as the mean ± SDs. Statistical notation: ** *p* < 0.01. (**A**–**C**), Two-way ANOVA followed by Tukey’s multiple comparisons test.

Rotarod fatigue testing was conducted in 12-week-old *Clock*^Δ19^ and wild-type (WT) mice at two circadian phases: ZT0 (rest phase) and ZT12 (activity onset). The results showed that *Clock*^Δ19^ mice exhibited significantly shorter retention times on the rotarod compared to WT mice at both time points, indicating increased susceptibility to fatigue. Notably, the difference was statistically significant only at ZT0, suggesting that *Clock*^Δ19^ mice are particularly prone to fatigue when exercised during their rest phase (Figure 2A). In contrast, no significant differences in endurance capacity were observed between *Clock*^Δ19^ and WT mice at either time point, as measured by total running distance until exhaustion (Figure 2B) and the number of electric shocks received within specific time intervals (Figure 2C). These results indicate that overall exercise tolerance was comparable across genotypes.

### 3.3. Clock^Δ19^ Mutation Abolishes Exercise-Induced Circadian Variations in Blood Glucose and Triglyceride Metabolism

**Figure 3:**
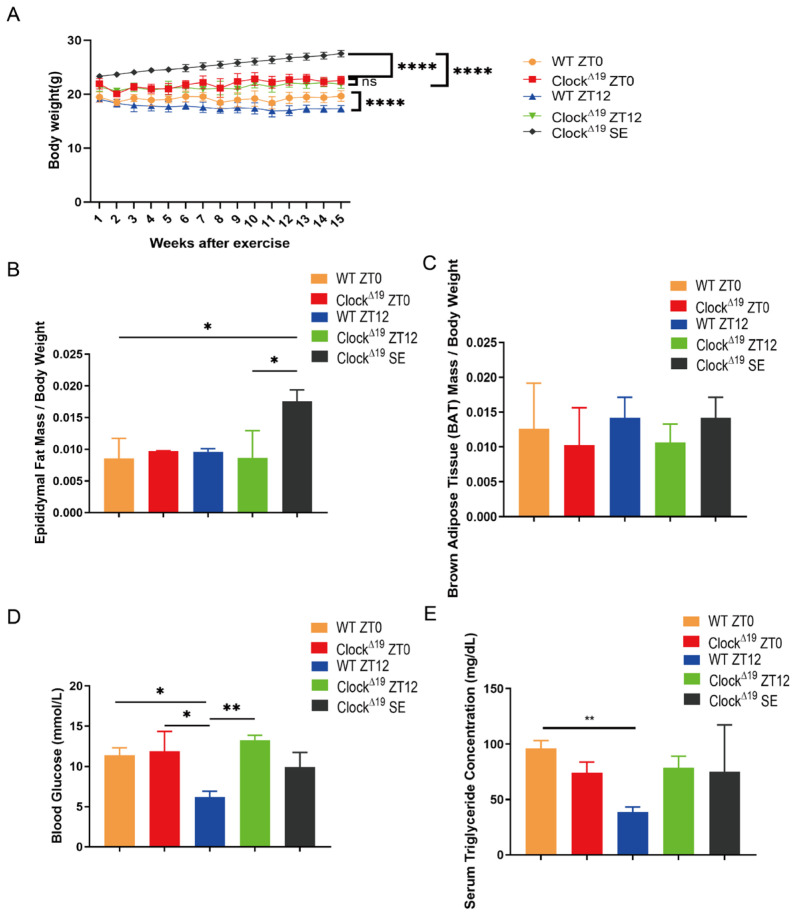
Exercise challenge unmasks time-dependent metabolic defects in *Clock*^Δ19^ mice. (**A**) Body weight progression curves of five experimental groups over 15-week period: ● WT ZT0, ■ WT ZT12, ▲ *Clock*^Δ19^ ZT0, ▼ *Clock*^Δ19^ ZT12, ◆ *Clock*^Δ19^ SE (sedentary control) (*n* = 5 per group). (**B**) Ratio of peri-epididymal fat to body weight of five groups (*n* = 5 per group). (**C**) Fasting blood glucose concentrations in five experimental groups (*n* = 5 per group). (**D**) Serum triglyceride concentrations in five experimental groups (*n* = 5 per group). The data are presented as the mean ± SDs. Statistical notation: * *p* < 0.05, ** *p* < 0.01, **** *p* < 0.0001, ns, not significant (p≥0.05). (**A**), Two-way ANOVA followed by Tukey’s multiple comparisons test; (**B**–**E**) One-way ANOVA followed by Tukey’s multiple comparisons test.

To explore the underlying mechanisms of increased fatigue susceptibility in *Clock*^Δ19^ mice, we conducted longitudinal metabolic assessments over the 15-week intervention period. Weekly body mass measurements revealed distinct responses to time-of-day-specific exercise (Figure 3A). At baseline, *Clock*^Δ19^ mice weighed more than age-matched wild-type (WT) controls, consistent with their known metabolic impairments. In WT mice, body weight began to diverge between ZT0 and ZT12 exercise groups after three weeks of training, with these differences becoming more pronounced over time. In contrast, *Clock*^Δ19^ mice showed no significant differences in body weight reduction between ZT0 and ZT12 exercise groups (*p* > 0.05). However, both exercised *Clock*^Δ19^ groups (ZT0 and ZT12) exhibited significant weight reduction compared to *Clock*^Δ19^ sedentary controls (SE) (*p* < 0.0001), indicating that endurance exercise effectively counteracts circadian disruption-induced weight gain independent of temporal alignment.

Further analysis showed that sedentary mice accumulated more epididymal white adipose tissue (eWAT) than exercised mice, regardless of genotype (Figure 3B). However, the ratio of brown adipose tissue mass to body weight showed no significant differences between the five experimental groups (Figure 3C). Exercise timing did not differentially affect fat accumulation, indicating that the reduction in adiposity was independent of circadian phase. Notably, WT mice displayed time-of-day-dependent fluctuations in fasting glucose and triglyceride concentrations (WT ZT0 vs. WT ZT12), patterns that were absent in *Clock*^Δ19^ mutants (Figure 3D,E).

### 3.4. Time-Dependent Endurance Exercise Improves Lipid Metabolism and Insulin Sensitivity in a Clock-Dependent Manner

**Figure 4:**
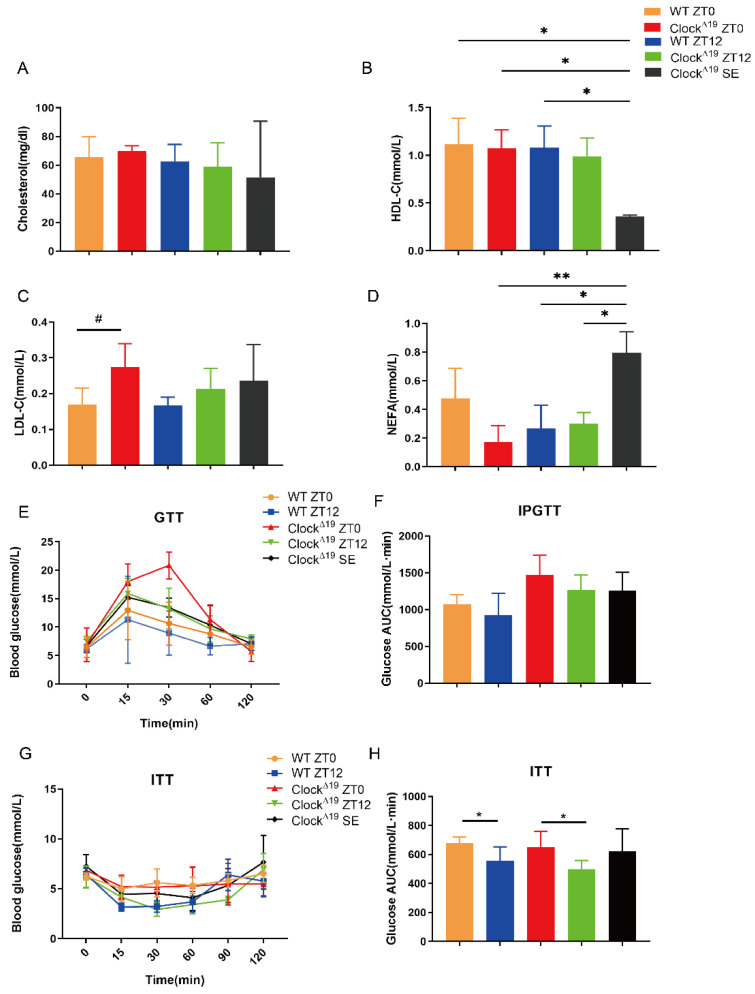
Long-term endurance exercise at ZT12 enhances HDL-C concentrations and insulin sensitivity in *Clock*^Δ19^ mice. (**A**) Serum total cholesterol concentrations in five experimental groups: ● WT ZT0, ■ WT ZT12, ▲ *Clock*^Δ19^ ZT0, ▼ *Clock*^Δ19^ ZT12, ◆ *Clock*^Δ19^ SE (sedentary control) (*n* = 5 per group). (**B**) Serum high-density lipoprotein cholesterol (HDL-C) concentrations in five experimental groups. (**C**) Serum low-density lipoprotein cholesterol (LDL-C) concentrations in five experimental groups, # *p* = 0.06. (**D**) Serum non-esterified fatty acid (NEFA) concentrations in five experimental groups. (**E**) Blood glucose concentrations (mmol/L) during the intraperitoneal glucose tolerance test (IPGTT). (**F**) Area under the curve (AUC) during a 120 min IPGTT. (**G**) Blood glucose concentrations (mmol/L) during the insulin tolerance test (ITT). (**H**) Area under the curve (AUC) during a 120 min ITT. The data are presented as the mean ± SDs. Statistical notation: * *p* < 0.05, ** *p* < 0.01. (**A**–**D**,**F**,**H**), One-way ANOVA followed by Tukey’s multiple comparisons test; (**E**,**G**) Two-way ANOVA followed by Tukey’s multiple comparisons test.

High-density lipoprotein cholesterol (HDL-C) plays a key cardioprotective role by facilitating reverse cholesterol transport, enabling the liver to clear excess cholesterol from peripheral tissues. We first assessed serum total cholesterol (TC) concentrations and found no significant differences among any experimental groups (Figure 4A), suggesting that general cholesterol homeostasis was maintained across conditions despite circadian or exercise interventions. In our study, endurance training significantly improved HDL-C concentrations in *Clock*^Δ19^ mice compared to their sedentary counterparts (Figure 4B), indicating a partial restoration of lipid homeostasis despite underlying circadian disruption. *Clock*^Δ19^ mutants also exhibited elevated low-density lipoprotein cholesterol (LDL-C) concentrations relative to WT mice, with no differences observed between ZT0 and ZT12 exercise groups (Figure 4C), indicating that LDL-C regulation was unaffected by exercise timing. In contrast, serum non-esterified fatty acids (NEFA) followed a circadian pattern and were reduced in exercised mice compared to sedentary controls (Figure 4D), consistent with known diurnal lipid cycling.

To further evaluate the impact of chronic exercise on metabolic health, intraperitoneal glucose tolerance tests (IPGTTs) and insulin tolerance tests (ITTs) were performed. After two weeks of training, no significant differences in glucose tolerance were observed between exercised (*Clock*^Δ19^ ZT0 and ZT12) and sedentary mice (Figure 4E,F). However, at the four-week mark, ITTs revealed clear improvements dependent on exercise timing (Figure 4G,H). Insulin sensitivity was significantly greater in both wild-type and *Clock*^Δ19^ mice exercising at ZT12 compared to their ZT0 counterparts. These results indicate that long-term endurance exercise aligned with the active phase (ZT12) enhances insulin sensitivity regardless of circadian status.

Taken together, these findings demonstrate that circadian-aligned endurance training improves systemic lipid metabolism by enhancing HDL-C concentrations and reducing NEFA, while LDL-C remains unchanged. Furthermore, exercise performed at the onset of the active phase (ZT12) rescues insulin sensitivity independently of core clock function. This highlights the potential of timed exercise to partially counteract lipid dysregulation and insulin resistance associated with circadian disruption.

## 4. Discussion

This study offers important insights into the relationship between circadian rhythms and long-term endurance exercise, revealing both clock-dependent and independent mechanisms that drive metabolic adaptations. Our results show that exercise timing profoundly influences metabolic outcomes in wild-type (WT) mice, with nocturnal exercise (ZT12) significantly improving lipid metabolism, insulin sensitivity, and glucose tolerance. These timing-specific benefits were absent in *Clock*^Δ19^ mice, highlighting the essential role of an intact circadian clock in optimizing the effects of exercise. Nonetheless, exercise universally increased HDL-C concentrations and reduced adiposity across both genotypes, indicating that some metabolic benefits of exercise occur independently of circadian regulation. These findings both support and challenge current models in chronobiology and exercise physiology, offering new avenues for research and clinical application.

Our results align with previous studies demonstrating circadian modulation of exercise performance and metabolic responses [17,18]. Research in rodents and humans has documented daily fluctuations in exercise capacity linked to factors such as energy substrate availability and clock gene expression [19,20]. The enhanced metabolic effects of ZT12 exercise in WT mice correspond with evidence that activity during the active phase maximizes substrate use and mitochondrial function [21,22]. However, these findings diverge from some human studies advocating morning exercise to improve fat oxidation. A critical synthesis of these discrepancies is warranted: differences in exercise modality (endurance vs. resistance), species-specific diurnal preferences (nocturnal rodents vs. diurnal humans), feeding schedules, and genetic backgrounds likely contribute to the observed variability. Moreover, the distinction between acute post-exercise responses and long-term adaptations may explain why some human trials report conflicting outcomes when exercise timing is not maintained consistently over weeks [23,24,25]. The absence of timing effects in *Clock*^Δ19^ mice supports the role of peripheral clocks, particularly in skeletal muscle, in mediating time-dependent exercise benefits [26,27].

Critically, exercised *Clock*^Δ19^ groups exhibited significantly reduced body weight alongside improved lipid homeostasis—including higher HDL-C and lower triglycerides—compared to sedentary controls, yet showed no differential responses between ZT0 and ZT12 cohorts. This concurrent amelioration of adiposity and dyslipidemia demonstrates that endurance exercise can partially counteract core circadian disruption. However, the precise molecular pathways underlying these effects were not directly interrogated in our study. Therefore, the following mechanistic interpretation should be considered hypothetical and as a basis for future investigation: reduced adipose mass may enhance lipid clearance through diminished ectopic fat deposition and elevated adiponectin signaling, thereby enabling PPARα-mediated induction of fatty acid oxidation genes and HDL-driven reverse cholesterol transport. While this hypothesis is consistent with previous literature, it remains to be tested in our model using tissue-specific knockouts or pharmacological interventions. Importantly, the correlation between the magnitude of weight loss and HDL-C elevation observed in our study is consistent with a potential causal role of adipose reduction, but does not establish causation. Taken together, endurance exercise in *Clock*^Δ19^ mice induces timing-independent metabolic improvements that likely involve multiple converging pathways, with adipose-lipid axis remodeling as one plausible contributor. Our data show that *Clock*^Δ19^ mice retain partial metabolic benefits (e.g., weight loss, HDL-C increase) but lack the timing-specific enhancements seen in WT mice. For instance, insulin sensitivity improved only with ZT12 exercise in both genotypes, yet the magnitude of improvement was attenuated in *Clock*^Δ19^ mice (Figure 4G,H). Thus, instead of full compensation, the results indicate a partial, timing-independent rescue of certain metabolic parameters. This nuanced view is essential for accurate interpretation.

The dual nature of exercise effects, both circadian-dependent and circadian-independent, has important implications for individuals with disrupted rhythms, such as shift workers, older adults, and patients with metabolic syndrome [28,29]. For those with intact circadian systems, exercising during the active phase may enhance metabolic benefits, as demonstrated in wild-type mice [23]. In contrast, individuals with circadian misalignment may still gain some advantages from exercise, though without the added benefit of timing specificity. This distinction highlights the potential for personalized exercise programs tailored to an individual’s circadian profile.

A key debate in the field concerns whether the timing of exercise or its consistency is more critical for metabolic health [30]. While our findings emphasize the importance of timing in wild-type mice, the universal benefits observed in *Clock*^Δ19^ mice suggest that regular exercise alone may suffice in circadian-disrupted conditions. However, a major confounder in circadian exercise studies is feeding timing. In the present experimental design, food was strictly restricted to the dark phase (ZT12–ZT24) for all groups. This controlled for one major variable, but it also raises the possibility that the observed benefits of ZT12 exercise could be partly attributed to the proximity of exercise to the onset of the feeding period. In rodents, physical activity can interact with meal timing to potentiate nutrient partitioning [30]. Under free-feeding conditions or when meal timing is misaligned, the time-of-day effects of exercise may differ. Therefore, future studies should systematically vary exercise timing and feeding timing independently to dissociate these factors. Disentangling the relative contributions of exercise timing and feeding timing will require further investigation.

Finally, the question of whether central or peripheral clocks primarily mediate exercise benefits persists. The metabolic improvements seen in *Clock*^Δ19^ mice, which have disrupted central and peripheral clocks, suggest that alternative pathways such as hormonal or neuronal signals may partially compensate. However, because the *Clock*^Δ19^ mutation affects all tissues; therefore, tissue-specific rescue or knockout strategies will be essential to dissect the relative contributions of central versus peripheral clocks to timed exercise adaptations.

## 5. Conclusions

This study deepens our understanding of the interplay between circadian rhythms and exercise in regulating metabolic health. We show that the full benefits of time-specific exercise depend on an intact circadian clock, while certain metabolic improvements can still occur through clock-independent mechanisms. These findings suggest that aligning physical activity with endogenous circadian rhythms may optimize metabolic outcomes in systems with preserved clock function. However, given the preclinical nature of this work (mouse model), direct extrapolation to human clinical practice is not yet warranted.

The concept of “chrono-exercise”—scheduling exercise according to an individual’s circadian phase—is supported by the present data as a hypothesis requiring further validation in humans. The observation that *Clock*^Δ19^ mice retain some timing-independent benefits does not diminish the importance of circadian alignment; rather, it underscores the need for translational studies that carefully account for feeding schedules, genetic backgrounds, and species differences.

In summary, this study provides a preclinical foundation for optimizing exercise strategies based on circadian principles. Future research should focus on: (1) testing the adipose-lipid axis hypothesis using tissue-specific interventions; (2) dissociating exercise timing from feeding timing in controlled human trials; and (3) evaluating whether the timing-dependent benefits observed in mice are reproducible in shift workers or other populations with circadian disruption. While the concept of circadian-aligned exercise holds promise, its clinical utility remains to be established. The present findings should therefore be interpreted with caution when extrapolated to human applications.

## Data Availability

The dataset supporting the conclusions of this article is included within the article.

## References

[1] Bass J., Lazar M.A. (2016). Circadian time signatures of fitness and disease. Science.

[2] Challet E. (2019). The circadian regulation of food intake. Nat. Rev. Endocrinol..

[3] Martin R.A., Viggars M.R., Esser K.A. (2023). Metabolism and exercise: The skeletal muscle clock takes centre stage. Nat. Rev. Endocrinol..

[4] Takahashi J.S. (2016). Transcriptional architecture of the mammalian circadian clock. Nat. Rev. Genet..

[5] Gabriel B.M., Zierath J.R. (2019). Circadian rhythms and exercise—Re-setting the clock in metabolic disease. Nat. Rev. Endocrinol..

[6] Petrenko V., Sinturel F., Riezman H., Dibner C. (2023). Lipid metabolism around the body clocks. Prog. Lipid Res..

[7] Stenvers D.J., Scheer F.A.J.L., Schrauwen P., la Fleur S.E., Kalsbeek A. (2018). Circadian clocks and insulin resistance. Nat. Rev. Endocrinol..

[8] Kanaley J.A., Porter J.W., Winn N.C., Lastra G., Chockalingam A., Pettit-Mee R.J., Petroski G.F., Cobelli C., Schiavon M., Parks E.J. (2023). Temporal optimization of exercise to lower fasting glucose levels. J. Physiol..

[9] Moholdt T., Parr E.B., Devlin B.L., Debik J., Giskeødegård G., Hawley J.A. (2021). The effect of morning vs evening exercise training on glycaemic control and serum metabolites in overweight/obese men: A randomised trial. Diabetologia.

[10] Peek C.B., Levine D.C., Cedernaes J., Taguchi A., Kobayashi Y., Tsai S.J., Bonar N.A., McNulty M.R., Ramsey K.M., Bass J. (2017). Circadian Clock Interaction with HIF1α Mediates Oxygenic Metabolism and Anaerobic Glycolysis in Skeletal Muscle. Cell Metab..

[11] Haganes K.L., Silva C.P., Eyjólfsdóttir S.K., Steen S., Grindberg M., Lydersen S., Hawley J.A., Moholdt T. (2022). Time-restricted eating and exercise training improve HbA1c and body composition in women with overweight/obesity: A randomized controlled trial. Cell Metab..

[12] Sato S., Dyar K.A., Treebak J.T., Jepsen S.L., Ehrlich A.M., Ashcroft S.P., Trost K., Kunzke T., Prade V.M., Small L. (2022). Atlas of exercise metabolism reveals time-dependent signatures of metabolic homeostasis. Cell Metab..

[13] Chen J., Xiang J., Zhou M., Huang R., Zhang J., Cui Y., Jiang X., Li Y., Zhou R., Xin H. (2025). Dietary timing enhances exercise by modulating fat-muscle crosstalk via adipocyte AMPKα2 signaling. Cell Metab..

[14] Sato S., Basse A.L., Schönke M., Chen S., Samad M., Altıntaş A., Laker R.C., Dalbram E., Barrès R., Baldi P. (2019). Time of Exercise Specifies the Impact on Muscle Metabolic Pathways and Systemic Energy Homeostasis. Cell Metab..

[15] Beals J.W., Kayser B.D., Smith G.I., Schweitzer G.G., Kirbach K., Kearney M.L., Yoshino J., Rahman G., Knight R., Patterson B.W. (2023). Dietary weight loss-induced improvements in metabolic function are enhanced by exercise in people with obesity and prediabetes. Nat. Metab..

[16] Turek F.W., Joshu C., Kohsaka A., Lin E., Ivanova G., McDearmon E., Laposky A., Losee-Olson S., Easton A., Jensen D.R. (2005). Obesity and Metabolic Syndrome in Circadian Clock Mutant Mice. Science.

[17] Savikj M., Stocks B., Sato S., Caidahl K., Krook A., Deshmukh A.S., Zierath J.R., Wallberg-Henriksson H. (2022). Exercise timing influences multi-tissue metabolome and skeletal muscle proteome profiles in type 2 diabetic patients—A randomized crossover trial. Metabolism.

[18] Iwayama K., Kurihara R., Nabekura Y., Kawabuchi R., Park I., Kobayashi M., Ogata H., Kayaba M., Satoh M., Tokuyama K. (2015). Exercise Increases 24-h Fat Oxidation Only When It Is Performed Before Breakfast. EBioMedicine.

[19] Smith H.A., Templeman I., Davis M., Slater T., Clayton D.J., Varley I., James L.J., Middleton B., Johnston J.D., Karagounis L.G. (2024). Characterizing 24-Hour Skeletal Muscle Gene Expression Alongside Metabolic and Endocrine Responses Under Diurnal Conditions. J. Clin. Endocrinol. Metab..

[20] Ezagouri S., Zwighaft Z., Sobel J., Baillieul S., Doutreleau S., Ladeuix B., Golik M., Verges S., Asher G. (2019). Physiological and Molecular Dissection of Daily Variance in Exercise Capacity. Cell Metab..

[21] Xin H., Huang R., Zhou M., Chen J., Zhang J., Zhou T., Ji S., Liu X., Tian H., Lam S.M. (2023). Daytime-restricted feeding enhances running endurance without prior exercise in mice. Nat. Metab..

[22] Chalom M.M., Lee C.-H. (2023). Time-restricted feeding makes the mouse run like a pro. Nat. Metab..

[23] Schumacher L.M., Thomas J.G., Raynor H.A., Rhodes R.E., Bond D.S. (2020). Consistent Morning Exercise May Be Beneficial for Individuals with Obesity. Exerc. Sport Sci. Rev..

[24] Creasy S.A., Wayland L., Panter S.L., Purcell S.A., Rosenberg R., Willis E.A., Shiferaw B., Grau L., Breit M.J., Bessesen D.H. (2022). Effect of Morning and Evening Exercise on Energy Balance: A Pilot Study. Nutrients.

[25] Chacko E. (2024). Morning exercise as fasted-state activity. Diabetologia.

[26] Kumar A., Vaca-Dempere M., Mortimer T., Deryagin O., Smith J.G., Petrus P., Koronowski K.B., Greco C.M., Segalés J., Andrés E. (2024). Brain-muscle communication prevents muscle aging by maintaining daily physiology. Science.

[27] Kelu J.J., Hughes S.M. (2025). Muscle peripheral circadian clock drives nocturnal protein degradation via raised Ror/Rev-erb balance and prevents premature sarcopenia. Proc. Natl. Acad. Sci. USA.

[28] Dieli-Conwright C.M., Courneya K.S., Demark-Wahnefried W., Sami N., Lee K., Buchanan T.A., Spicer D.V., Tripathy D., Bernstein L., Mortimer J.E. (2018). Effects of Aerobic and Resistance Exercise on Metabolic Syndrome, Sarcopenic Obesity, and Circulating Biomarkers in Overweight or Obese Survivors of Breast Cancer: A Randomized Controlled Trial. J. Clin. Oncol..

[29] Sandsdal R.M., Juhl C.R., Jensen S.B.K., Lundgren J.R., Janus C., Blond M.B., Rosenkilde M., Bogh A.F., Gliemann L., Jensen J.-E.B. (2023). Combination of exercise and GLP-1 receptor agonist treatment reduces severity of metabolic syndrome, abdominal obesity, and inflammation: A randomized controlled trial. Cardiovasc. Diabetol..

[30] Li Y., Pan A., Wang D.D., Liu X., Dhana K., Franco O.H., Kaptoge S., Di Angelantonio E., Stampfer M., Willett W.C. (2018). Impact of Healthy Lifestyle Factors on Life Expectancies in the US Population. Circulation.

